# The VICTORY (Investigation of Inflammacheck to Measure Exhaled Breath Condensate Hydrogen Peroxide in Respiratory Conditions) Study: Protocol for a Cross-sectional Observational Study

**DOI:** 10.2196/23831

**Published:** 2021-07-09

**Authors:** Lauren Fox, Jessica Gates, Ruth De Vos, Laura Wiffen, Alexander Hicks, Hitasha Rupani, Jane Williams, Thomas Brown, Anoop J Chauhan

**Affiliations:** 1 Portsmouth Hospitals University NHS Trust Portsmouth United Kingdom; 2 University Hospital Southampton NHS Trust Southampton United Kingdom; 3 Equine Department Hartpury University Gloucestershire United Kingdom; 4 Faculty of Science and Health University of Portsmouth Portsmouth United Kingdom

**Keywords:** medical device, diagnosis, hydrogen peroxide, lung diseases, exhalation, asthma, COPD, bronchiectasis, interstitial lung disease, lung cancer, breathing pattern disorder, pneumonia

## Abstract

**Background:**

More than 7% of the world’s population is living with a chronic respiratory condition. In the United Kingdom, lung disease affects approximately 1 in 5 people, resulting in over 700,000 hospital admissions each year. People with respiratory conditions have several symptoms and can require multiple health care visits and investigations before a diagnosis is made. The tests available can be difficult to perform, especially if a person is symptomatic, leading to poor quality results. A new, easy-to-perform, point-of-care test that can be performed in any health care setting and that can differentiate between various respiratory conditions would have a significant, beneficial impact on the ability to diagnose respiratory diseases.

**Objective:**

The objective of this study is to use a new handheld device (Inflammacheck) in different respiratory conditions to measure the exhaled breath condensate hydrogen peroxide (EBC H_2_O_2_) and compare these results with those of healthy controls and with each other. This study also aims to determine whether the device can measure other parameters, including breath humidity, breath temperature, breath flow dynamics, and end tidal carbon dioxide.

**Methods:**

We will perform a single-visit, cross-sectional observational study of EBC H_2_O_2_ levels, as measured by Inflammacheck, in people with respiratory disease and volunteers with no known lung disease. Participants with a confirmed diagnosis of asthma, chronic obstructive pulmonary disease, lung cancer, bronchiectasis, pneumonia, breathing pattern disorder, and interstitial lung disease as well as volunteers with no history of lung disease will be asked to breathe into the Inflammacheck device to record their breath sample.

**Results:**

The results from this study will be available in 2022, in anticipation of COVID-19–related delays.

**Conclusions:**

This study will investigate the EBC H_2_O_2_, as well as other exhaled breath parameters, for use as a future diagnostic tool.

## Introduction

### Respiratory Diseases

Globally, respiratory disease is a leading cause of morbidity and mortality, with over 7% of the world’s population living with a chronic respiratory disease [1], including a significant burden on children. Respiratory diseases account for more than 10% of all disability-adjusted life years worldwide [2]. Diagnosing respiratory conditions at an earlier stage affords patients the opportunity to receive appropriate treatment, along with reducing symptoms and improving quality of life and reducing the impact of frequent visits to health care services due to misdiagnosis or exacerbations. A rapid point-of-care test may be more useful in low-resource settings.

Within the United Kingdom, approximately 1 in 5 people have ever developed asthma, chronic obstructive pulmonary disease (COPD), or other long-term respiratory illness [3]. In addition, lung diseases are responsible for more than 700,000 hospital admissions and over 6 million inpatient bed days each year [3]. It is estimated that each week, 10,000 people are given a new diagnosis of lung disease, but there are thousands of more people living with the symptoms of lung conditions without a diagnosis.

Table 1 presents the respiratory conditions that are under investigation in this study. As the table shows, there is no one gold standard diagnostic investigation to support the diagnosis of these different conditions. As a consequence, patients can undergo multiple investigations, leading to a delay in diagnosis and a prolonged period of struggling with untreated symptoms.

**Table 1:** Disease group summaries and diagnostic investigations.

Respiratory disease	Background (key facts)	Diagnostic investigations
COPD^a^	In the United Kingdom, there are an estimated 3 million people affected by COPD, with a third of those having a diagnosis and two-thirds who remain undiagnosed [4]. COPD is common and treatable but is a leading cause of morbidity and mortality worldwide, and its prevalence is likely to increase in the next decade [5]. COPD is responsible for a significant financial burden on the NHS^b^, with the total annual cost of COPD reaching £982 million (US $1.3 billion), as well as the personal burden, causing almost 22 million lost working days per year [6].	Spirometry, chest x-ray, and CT^c^ scan
Asthma	Asthma affects over 5.4 million people in the United Kingdom, with 200,000 experiencing severe disease with frequent exacerbations [7]. The National Review of Asthma Deaths in 2014 found that there were 900 deaths due to asthma, highlighting that more needs to be done regarding the diagnosis and management of this disease [8]. Treating asthma costs the NHS over £1 billion (US $1.42 billion) per year, and it is a burden for the person living with asthma, with significant psychological and physical morbidity [9,10]. Delays in diagnosing asthma and achieving good disease control cause an increase in emergency health care use and inappropriate treatment.	Peak expiratory flow, spirometry, bronchial provocation, exhaled nitric oxide, and eosinophilic markers
Bronchiectasis	The prevalence of bronchiectasis in the United Kingdom is rising, and it is estimated that the number of people living with this disease exceeds 300,000 [11,12]. People with bronchiectasis experience symptoms of excessive sputum production and recurrent respiratory infections, sometimes requiring hospital admission.	Chest x-ray, CT scan, and sputum microbiology
ILD^d^	ILDs are a group of diffuse parenchymal lung disorders associated with substantial morbidity and mortality. ILD can be difficult to diagnose and often requires collaborative expertise from multiple specialties to reach a consensus. As a consequence, ILD is often diagnosed late in the course of the disease, impacting patients’ quality of life, and can even limit access to novel treatments [13,14]. For example, idiopathic pulmonary fibrosis, one of the more common ILDs, is a chronic, progressive disease with a median survival of only 3 years from the time of diagnosis [15]. As such, developing a test that could confirm ILD at an earlier stage is urgently required.	CT scan or lung biopsy
Lung cancer	Lung cancer is a leading cause of global cancer death [16]. Even with recent advances in treatment options, approximately 86% of lung cancer patients die within 5 years of diagnosis. However, with early detection and treatment, 5-year survival rates improve to 70% in patients with stage 1 disease [17]. Consequently, new, accurate diagnostic tests that can detect lung cancer at the earliest stage possible are urgently needed.	CT scan and lung biopsy
Pneumonia	Pneumonia is the most common cause of respiratory hospital admissions. It is the inflammation of one or both lungs, normally caused by an infection. It is estimated that approximately 220,000 people receive a diagnosis of pneumonia each year [3]. Pneumonia is responsible for over 5% of all deaths and over 25% of all respiratory deaths [3]. It can be difficult to diagnose, and diagnosis can depend upon the quality of the chest x-ray or the ability of the person to mount an immune response.	Chest x-ray and CT scan
BPD^e^	BPD is a very common, potentially debilitating, and easily treatable condition. Unfortunately, it is rarely considered as a common cause of breathlessness, and besides the Nijmegen questionnaire, there is no quick and easy way of diagnosis [18].	Clinical assessment

^a^COPD: chronic obstructive pulmonary disease.

^b^NHS: National Health Service.

^c^CT: computed tomography.

^d^ILD: interstitial lung disease.

^e^BPD: breathing pattern disorder.

### Airway and Lung Inflammation

A common feature of most of these lung conditions is airway inflammation. The current breathing devices to measure the levels of inflammation focus on eosinophilic rather than neutrophilic inflammation, which is the predominant phenotype in COPD, bronchiectasis, steroid-resistant asthma, and pneumonia. Prolonged exposure to toxic irritants has been implicated in airway inflammation in lung cancer [19]. Interstitial lung disease (ILD) is an umbrella term for lung diseases characterized by fibrosis, which can occur as a result of chronic inflammation, remodeling, and repair [20].

Airway inflammation leads to an imbalance between the production of reactive oxygen species (ROS) and the ability of the body to counteract their harmful effects through neutralization by antioxidants. This results in oxidative stress [21,22]. Increased expression of ROS by activated inflammatory cells, including neutrophils, macrophages, and eosinophils, can lead to further generation of inflammatory mediators, causing damage to epithelial cells and increased bronchial hyperreactivity. ROS are metabolized in cells to produce highly reactive oxidants such as hydrogen peroxide (H_2_O_2_), which is fat soluble and can move across cell membranes. As H_2_O_2_ is volatile and readily equilibrates with air, its presence is detected in exhaled breath condensate (EBC). Therefore, measurement of exhaled breath condensate hydrogen peroxide (EBC H_2_O_2_) provides a quantitative measure of oxidative stress and airway or lung inflammation [23,24].

Breathing pattern disorder (BPD) is a noninflammatory condition but is a common comorbidity with other respiratory disorders. Preliminary data have shown that breath flow dynamics in a person with BPD is unique (D. Neville, unpublished data, 2018); therefore, by studying those with BPD only, we may be able to identify specific features and use the device to assess when dual pathology is present.

### Inflammacheck

In contrast to the current measures of airway inflammation, the collection of EBC H_2_O_2_ is performed during tidal breathing, making it noninvasive and easy to perform. It can be repeated quickly and is well tolerated even in patients with severe airway disease. It is widely appreciated that EBC H_2_O_2_ measurement has the potential to improve clinical practice by safely providing vital information on aspects of disease that are currently inaccessible [25]. To date, measurement of H_2_O_2_ in EBC requires complex, multistep processing of the collected breath samples to produce a result, and consequently, it has largely been used as a research tool.

**Figure 1:**
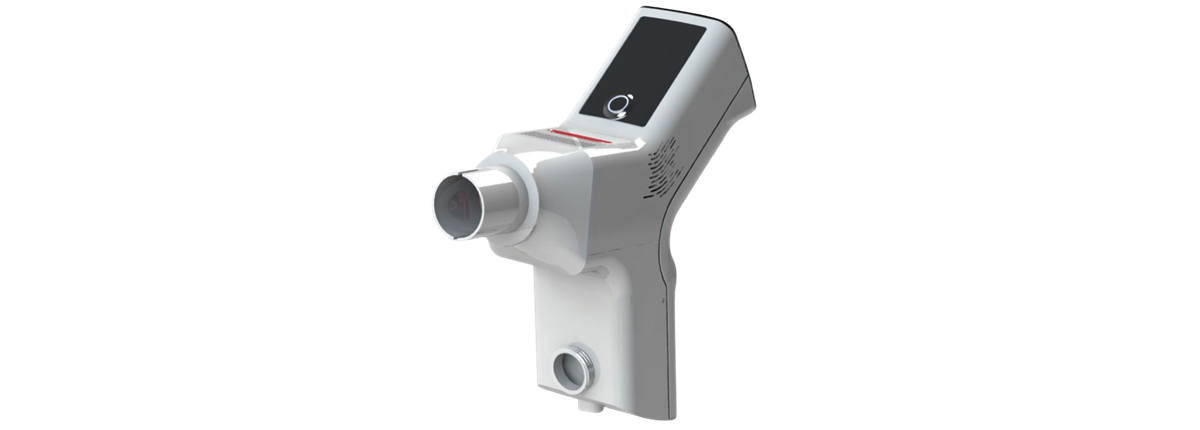
The Inflammacheck device.

Exhalation Technology Ltd developed a table-top device for point-of-care H_2_O_2_ measurement in exhaled breath—the *Inflammacheck* device. Previous studies including EXHALE 1A (Exhaled Hydrogen Peroxide as a Marker of Lung Disease Study 1–Airways; A. Chauhan, unpublished data, 2019) and EXHALE 1B (Exhaled Hydrogen Peroxide as a Marker of Lung Disease Study 1–Breathlessness; [26]) have shown that it accurately measures the level of H_2_O_2_ in EBC and that levels of H_2_O_2_ are significantly higher in patients with asthma, COPD, ILD, and lung cancer than in healthy controls. The EXHALE 1V (Exhaled Hydrogen Peroxide as a Marker of Lung Disease Study 1–Validation) COPD validation study was terminated early as significant changes were made in the sensor processing and device housing, which made the results unreliable. Participant feedback from these studies helped refine the device further, and now, Exhalation Technology Ltd has produced a handheld device (Figure 1), which can accurately and reliably measure the H_2_O_2_ level in a single test. This new device can also measure additional parameters, including breath humidity, breath temperature, breath flow dynamics, and carbon dioxide (CO_2_) waveform. Previous studies have demonstrated significant differences in H_2_O_2_ levels between healthy controls and some respiratory diseases including asthma, COPD, and ILD. We now want to expand upon these results to determine whether these differences are seen across a wider spectrum of respiratory diseases and whether the additional parameters measured by the Inflammacheck device (Exhalation Technology Ltd) also reflect differences between conditions. This could potentially provide a unique test that measures common biological and physiological parameters in a point-of-care device to aid earlier diagnosis and support personalized management plans. This simple, noninvasive technique may also make repeat sampling and longitudinal monitoring of global airway inflammation a realistic possibility. The Inflammacheck device is currently Conformité Européenne marked for adults only, which is the subject of this protocol.

### Objectives

#### Primary Objective

The primary objective is to measure and compare the levels of H_2_O_2_ in EBC in patients with a range of commonly occurring respiratory conditions and compare these results with those of healthy controls, and with each other.

#### Secondary Objectives

The secondary objectives are as follows:

- To assess whether the device can measure EBC H_2_O_2_ consistently (pilot phase only) and ensure that there are no safety issues or participant issues when using the device
- To determine whether EBC H_2_O_2_, as measured by Inflammacheck, can differentiate between patients with asthma, COPD, lung cancer, ILD, BPD, bronchiectasis, and pneumonia and healthy controls
- To measure and compare the levels of the following parameters using Inflammacheck in patients with a range of commonly occurring respiratory conditions and compare with healthy controls:
Breath humidity
  Breath temperature
  Exhaled CO_2_ waveform
  Breath flow dynamics
- To determine whether H_2_O_2_, breath humidity, breath temperature, exhaled CO_2_ waveform, and breath flow dynamics, as measured by Inflammacheck, can either alone or in combination differentiate patients with asthma, COPD, lung cancer, ILD, BPD, bronchiectasis, and pneumonia from each other and from healthy controls
- To assess the experience of a small group of participants and health care professionals in using the Inflammacheck device using a questionnaire methodology

## Methods

### Overview

This is a cross-sectional observational study of EBC H_2_O_2_ levels, as measured by Inflammacheck, in people with asthma, COPD, bronchiectasis, ILD, lung cancer, pneumonia, and BPD and in healthy volunteers. The participants were screened for the absence of COVID-19 symptoms before testing.

### Outcome Measures

#### Respiratory Outcomes

Respiratory outcomes will include the following:

- EBC H_2_O_2_ measured by Inflammacheck
- Breath humidity
- Breath temperature
- Exhaled CO_2_ waveform
- Breath flow dynamics
- Forced expiratory volume in 1 second (FEV_1_), forced vital capacity (FVC), and expiratory ratio as appropriate.

#### Experience Outcomes (by Questionnaire)

Experience outcomes will include the following:

- Rating of *ease of use* of the test
- Rating of whether the test is acceptable to the participant
- The participant’s perception of the device
- The health care professional’s experience of the device

### Eligibility Criteria

#### Study Participants

Participants with the abovementioned respiratory conditions and healthy volunteers will be recruited. There are some overarching inclusion and exclusion criteria, along with some specific respiratory condition criteria to ensure that each disease is well defined.

#### Inclusion Criteria

The participants must meet all the following criteria to be considered eligible for the study:

- Male or female aged ≥16 years
- Asthma (confirmed by spirometry or airway inflammation and function tests) with a smoking history of ≤10 pack-years
- COPD (confirmed by spirometry)
- Bronchiectasis (confirmed by computed tomography scan)
- ILD (confirmed by computed tomography scan and multidisciplinary team or specialist consensus)
- Primary lung cancer (confirmed diagnosis by histology or radiology*)* and not yet started significant treatment
- Current pneumonia (confirmed by chest imaging)
- BPD (specialist confirmed) with no other significant respiratory comorbidity
- Healthy controls (defined as no current clinical diagnosis of, or receiving treatment for, a lung disorder or other significant medical disorder)
- Willing and able to give informed consent for participation in the study
- In a stable state (at least 4 weeks after treatment of an exacerbation of their respiratory disease)

#### Exclusion Criteria

The participants may not enter the study if any of the following apply:

- Existing comorbidities that may prevent them from performing spirometry (for those with asthma and COPD or healthy controls) and no spirometry in the previous 12 months (at the discretion of the clinical investigator)
- Unable to comprehend the study, unable to provide informed consent, and unable to perform any of the study procedures
- Requirement of supplemental oxygen (participants with pneumonia only)

### Sampling and Sample Size

The sample size was based on the primary objective of comparing EBC H_2_O_2_ values between each of the study groups (patients with asthma, COPD, ILD, and lung cancer and controls) based on the results from previous studies (EXHALE 1A and EXHALE 1B), using an older version of the device. Sample sizes were calculated based on 5% significance and 80% power using a comparison of 2 means (two-tailed *t* test across independent groups) and using effect sizes based on the results of the previous studies.

Using these calculations, we will recruit the following:

- 30 people with COPD
- 35 people with asthma
- 25 people with ILD
- 40 people with lung cancer
- 50 healthy controls

The BPD, bronchiectasis, and pneumonia groups are exploratory, and no information exists to base their sample size on. Therefore, we will aim to recruit 25 people in each of these groups.

### Study Procedures

#### Recruitment

This is a multicenter study in which the identification of potential participants are being performed in respiratory outpatient clinics, inpatient wards across the hospital, and integrated specialist community clinics in primary care. Participants in our research database who have previously provided their consent to participate in future studies that adhere to the general data protection regulations will also be offered the opportunity to participate in this study. People with suspected lung cancer can be recruited before a confirmed diagnosis. If tests confirm that the participant does not have cancer, they can be entered into any other category if appropriate; otherwise, they will be considered a screen fail. Healthy volunteers will be recruited from National Health Service staff, our research database, friends and family of participants with respiratory disease, and students or staff at the University of Portsmouth.

Participants will be approached by their clinical care team, and those who express an interest will be provided with a participant information sheet (PIS), an opportunity to ask any questions, and a review with the research team either at that point in time or at a future convenient date and time.

#### Pilot Phase

Before starting full recruitment, we will run a pilot phase to ensure that the device can produce consistent and reliable results. Participants will be recruited as detailed above, and the study procedures will be identical. These data will be analyzed as soon as possible after collection to assess the quality and accuracy of the results. The criteria for termination of the pilot phase will be determined by the clinical, engineering, and manufacturing teams based on the quality of data, safety information, clinical training, and ease of use.

### Study Assessments

#### Participant Characteristics

Before other study procedures, the characteristics of participants, which are known to have a potential influence on respiratory test results, will be documented. These will include the following:

- Demographics (age, gender, and ethnicity)
- Categories of disease-specific medications that participants are taking
- Categories of other medical comorbidities

#### Respiratory Tests

The participants will then perform the EBC H_2_O_2_ level measurement performed by the Inflammacheck device, followed by spirometry if the participant has asthma or COPD or is a healthy control, unless this has been done in secondary care or by the research team within the last 12 months. Following this, a small subgroup of participants will answer a brief Likert-type questionnaire regarding their experience. For every Inflammacheck test, the following will be recorded:

- Whether the test was completed, and if not, the reason for this
- Any adverse events (AEs) related to the test that are reported by the participant or noted by the clinical staff during the test procedures.

#### Hydrogen Peroxide in Exhaled Breath Condensate

EBC H_2_O_2_ will be measured using the Inflammacheck device according to the manufacturer’s instructions. This involves simple relaxed tidal breathing for up to 6 minutes into a disposable mouthpiece attached to the handheld device, while wearing a nose clip. The Inflammacheck device then provides a reading of the EBC H_2_O_2_. The results will not be recorded in any clinical notes, as they are not intended to inform patient management decisions in this study. As this is a point-of-care test, this investigation can be performed in any clinical environment. Understandably, preventing cross-contamination in any era, but especially in this global coronavirus pandemic, is paramount, and this investigation is performed using a bacterial viral filter within the mouthpiece, with single-use disposable valve houses and sensors. The device itself is cleaned between each participant’s use according to the local cleaning guidelines for medical devices.

#### Spirometry

Spirometry (performed conforming to the American Thoracic Society and European Respiratory Society standards) will only be performed in patients with asthma and COPD or in healthy controls who have not undergone spirometry in the last year in secondary care or by the respiratory research team. Participants will inhale rapidly and completely from functional residual capacity, then exhale in an initial forced exhalation, and then continue exhalation until the end of the breath. FEV_1_ (measured in liters), FVC (measured in liters), and FEV_1_/FVC ratio will be recorded. FEV_1_ and FVC will be documented as absolute values and as a percentage of the predicted value (25). A minimum of 3 blows will be performed, with 2 blows within 100 milliliters or 5% of each other. Given the current coronavirus pandemic, spirometry is only being performed if it will impact clinical decisions.

#### Self-Completion Questionnaire

A brief self-completion questionnaire will be used in 20 randomly selected participants to evaluate their opinions on the Inflammacheck device on a Likert-type scale. Participants will be asked about ease of use, comfort during testing, and perception and satisfaction of Inflammacheck.

#### Health Care Professionals’ Self-Completion Questionnaire

A brief self-completed questionnaire will be used by 5 randomly selected health care professionals to evaluate their opinions on the Inflammacheck device on a Likert-type scale, as described above.

### Participant Withdrawal

#### Withdrawal Rules

Participants who are unable to perform the EBC H_2_O_2_ measurement with Inflammacheck or spirometry will not be withdrawn. The reason for failing to perform these tests will be documented in the case report form (CRF).

#### Safety Assessments: AE

##### Overview

An AE is any untoward medical occurrence in a participant taking part in a clinical trial, which does not necessarily have to have a causal relationship with the device under investigation. An AE can, therefore, be any unfavorable or unintended sign, symptom, or disease temporarily associated with the use of the device, whether or not this has a causal relationship with the device under investigation.

##### Recording and Reporting of AEs

There are not expected to be any AEs associated with the use of the Inflammacheck device, and no AEs were noted during any of the previous studies that involved this device. Only AEs that have a reasonable possibility of being attributable to the device and any other AE considered to be of clinical significance by the principal investigator (PI; eg, causing harm to the patient) will be recorded in the CRF and reported to the sponsor as per their guidelines. We will record all AEs that are observed during all respiratory test procedures as a study outcome. Any AEs that do occur and are considered by the PI to be related to the device will be expedited to the sponsor, research ethics committee (REC), and the device manufacturer within 7 days. Lists of the AEs will be provided to the sponsor when requested.

#### Data Collection and Management

Data collection will be performed by the research team comprising research doctors, nurses, and clinical research associates, all of whom have received appropriate training. Enrollment in the study will be documented in each participant’s medical notes if they are an inpatient. The data collection forms are as follows:

- CRF, including participant characteristics and respiratory test results confirming the underlying diagnosis
- Self-completed questionnaire for participants

The data will be entered into a secure electronic platform. Anonymized data will be shared electronically with Exhalation Technology Ltd for analysis and interpretation of the breath humidity, temperature, breath flow dynamics, and CO_2_ waveform data. The Inflammacheck results will be uploaded to a secure shared database to allow analysis and review of the collected data. Participants will consent to this, and no identifiable data will be shared.

#### Data Analysis

All participants with an EBC H_2_O_2_ result, as measured by Inflammacheck, will be analyzed. Subgroup analyses may be carried out if there are sufficient number of patients with particular characteristics, such as smokers and nonsmokers.

#### Analysis of End Points

##### Summary Statistics

Demographics or baseline characteristics of each study group will be produced, as well as an overall summary for all groups combined. Normally distributed continuous variables will be summarized by the mean and SD, whereas the median and IQR will be preferred for nonnormally distributed continuous variables. The number and percentage of subjects in each category will be recorded for categorical variables.

##### Primary Analysis

The primary analysis will be a comparison of EBC H_2_O_2_ values, as measured by the Inflammacheck device, between participants with the index disease and controls and with each other. It is expected that the EBC H_2_O_2_ will have a positively skewed distribution. To allow for the skewed distribution, one approach would be to analyze the data on a log-transformed scale and to compare between groups using analysis of variance, with post hoc tests performed to compare between pairs of groups. It is possible that there may be EBC H_2_O_2_ measurements below the lower detection limit, and thus, the previous approach may not be practical. If measurements are below the detection limit, nonparametric methods will be used. The Kruskal-Wallis test will be used to evaluate if differences exist between the 3 groups, with post hoc Mann-Whitney *U* analyses used to compare differences between pairs of groups. When comparing pairs of groups, a Bonferroni correction will be applied to allow for multiple testing.

##### Secondary Analyses

The association between the EBC H_2_O_2_ measurements and other parameters (breath temperature, breath humidity, breath flow dynamics, and CO_2_ waveforms) will be examined. Associations between EBC H_2_O_2_ and the continuous variables listed above will be examined using either Pearson or Spearman rank correlation (as appropriate).

The percentage of attempted tests that failed for each test will be recorded. The association between patient characteristics and this outcome will be examined. Assuming that patient characteristics are categorical in nature, the chi-square test or Fisher exact test will be used to examine associations with this outcome. Secondary analyses will be performed for all subjects combined and for each study group separately.

##### Exploratory Analysis

The relationship between EBC H_2_O_2_ and other lung function parameters with respiratory disease type will be further examined, assuming that EBC H_2_O_2_ and other factors are possible predictors of respiratory disease. Respiratory disease type, using the patient groups included in the study, will be considered as a binary outcome: healthy versus disease type, and the analysis will be performed using multivariable logistic regression. Receiver operator curve characteristic analyses will also be undertaken to assess the predictability of EBC H_2_O_2_ levels to differentiate between healthy volunteers and patients with respiratory disease. Further analyses will be performed to assess whether H_2_O_2_ alone or in combination with temperature, humidity, breath flow dynamics, and CO_2_ waveform characteristics can discriminate between healthy controls and those with a lung disorder using sensitivity, specificity, and receiver operating characteristic curves as well as using generalized linear models with post hoc least significant difference testing.

##### Procedure for Dealing With Missing, Unused, and Spurious Data

The analysis will include any measured data values, with missing values omitted from the analysis. No imputation of the missing data will be performed. These data will be examined for outlying values. Where possible, these will be retained in the data analysis, and their influence will be minimized by a data transformation or a nonparametric approach. If such approaches are not practical, the analysis of the primary outcome will be performed twice, with and without the outlying values.

#### Ethics

##### Participant Confidentiality

The study staff will ensure that participants’ anonymity is maintained. The participants will be identified only by initials and a participant’s ID number on the CRF and any electronic database. All documents will be stored securely and will only be accessible by the study staff and authorized personnel. The study will comply with the Data Protection Act 2018, which requires data to be anonymized as soon as it is practicable to do so.

##### Other Ethical Considerations

The study was approved by the Berkshire REC (reference 19/SC/0462) in August 2019. They reviewed and approved the protocol and all study-relevant material. Any changes to the protocol or relevant study documents will be approved by the sponsor. If an amendment is made that requires REC approval, as defined by the REC as a substantial amendment, the changes will not be instituted until the amendment has been reviewed and received approval or favorable opinion from the REC and research and development departments. A protocol amendment intended to eliminate an apparent immediate hazard to participants may be implemented immediately, providing that the REC is notified as soon as possible, and an approval is requested. Minor amendments as defined by REC as a nonsubstantial amendment may be implemented immediately, and the REC will be informed. All participants will have adequate time to consider participation in the study, as per the Good Clinical Practice guidelines.

Patients who are already enrolled in other research trials will be invited and allowed to participate in the study if they wish to do so. This was discussed with our patient and public involvement (PPI) representatives who felt that these patients should have the opportunity to participate in this study and should not be excluded. There is a possibility that this study reveals potential new, previously unknown disease pathology. This would be more likely to occur in *healthy controls*. If such a circumstance occurs, then the participant will be told about the results and immediately referred to the most appropriate National Health Service department for further review. With the participant’s consent, a letter will be written to their general practitioner explaining the findings.

Where possible, this study will be performed alongside an existing appointment to limit any extra burden to the participant, and all participants will receive a small compensation for travel costs. Participants in the pneumonia arm will be recruited while an inpatient and will not require any additional visits.

##### Informed Consent

It is the responsibility of the investigator, or a person designated by the investigator (if acceptable by local regulations), to obtain written informed consent from each person participating in this study, after adequate explanation of the aims, methods, anticipated benefits, and potential hazards of the study using the PIS.

Electronic consent will be obtained using the REDCap (Research Electronic Data Capture; Vanderbilt University) platform. Once participants have read the PIS, they will be asked to enter their signature (using their cursor or finger if on a tablet device). Participants will have the opportunity to download or print a copy of their consent form and the PIS. If a participant is unable to complete e-consent, they will have the opportunity to sign a paper consent form when they attend to complete the study visit.

The process for obtaining informed consent will be in accordance with REC guidance, Good Clinical Practice guidelines, and any other regulatory requirements that might be introduced. The PI or delegate and the participant shall both sign and date the informed consent form before the person can participate in the study.

The decision regarding participation in the study is entirely voluntary. The investigator or their nominee shall emphasize to them that consent regarding study participation may be withdrawn at any time without penalty or without affecting the quality or quantity of their future medical care or without loss of benefits to which the participant is otherwise entitled.

#### PPI Details

PPI in this study has been sought from patients with firsthand experience of living with a respiratory disease. Through face-to-face meetings, email, and telephone contact, we have discussed the concept, impact, and details of the study with our patient representatives from local groups. They have contributed to developing the key questions and setting our study objectives, ensuring that we answer questions relevant to people with respiratory diseases. They have helped design the questionnaire that will be used to record the participant experience of the device and the PIS.

## Results

This study has been receiving funding since 2019 and was approved by a REC in August 2019. As of May 2020, the pilot phase has been completed, and 78 participants enrolled into the VICTORY (Investigation of Inflammacheck to Measure Exhaled Breath Condensate Hydrogen Peroxide in Respiratory Conditions) study. The results from this study will be available in 2022, in anticipation of COVID-19–related delays.

## Discussion

An easy-to-use point-of-care device that can be used in any health care setting and can differentiate between a range of respiratory conditions would revolutionize the diagnosis of respiratory conditions. By improving our ability to confirm a correct diagnosis, we could initiate earlier treatments, reduce the burden of frequent health care visits, and improve patients’ quality of life. The Inflammacheck device is noninvasive, repeatable, and requires minimal participant effort, which are beneficial attributes for patients with respiratory conditions who often struggle with breathlessness. The VICTORY study will evaluate the Inflammacheck device, using EBC H_2_O_2_ alongside further exhaled breath parameters as a diagnostic tool in respiratory conditions.

## Abbreviations

AE: adverse event
BPD: breathing pattern disorder
CO2: carbon dioxide
COPD: chronic obstructive pulmonary disease
CRF: case report form
EBC H_2_0_2_: exhaled breath condensate hydrogen peroxide
EBC: exhaled breath condensate
EXHALE 1A: Exhaled Hydrogen Peroxide as a Marker of Lung Disease Study 1–Airways
EXHALE 1B: Exhaled Hydrogen Peroxide as a Marker of Lung Disease Study 1–Breathlessness
EXHALE 1V: Exhaled Hydrogen Peroxide as a Marker of Lung Disease Study 1–Validation
FEV1: forced expiratory volume in 1 second
FVC: forced vital capacity
H_2_0_2_: hydrogen peroxide
ILD: interstitial lung disease
PI: principal investigator
PIS: participant information sheet
PPI: patient and public involvement
REC: research ethics committee
REDCap: Research Electronic Data Capture
ROS: reactive oxygen species
VICTORY: Investigation of Inflammacheck to Measure Exhaled Breath Condensate Hydrogen Peroxide in Respiratory Conditions
